# Proline-Rich Protein Tyrosine Kinase 2 in Inflammation and Cancer

**DOI:** 10.3390/cancers10050139

**Published:** 2018-05-08

**Authors:** Xiangdong Zhu, Yonghua Bao, Yongchen Guo, Wancai Yang

**Affiliations:** 1 Institute of Precision Medicine, Jining Medical University, Jining 272067, China; baoyonghua2005@mail.jnmc.edu.cn (Y.B.); guoyongchen2005@mail.jnmc.edu.cn (Y.G.); 2 Department of Emergency Medicine, University of Illinois at Chicago, Chicago, IL 60612, USA; 3 Department of Pathology, University of Illinois at Chicago, Chicago, IL 60612, USA

**Keywords:** FAK, Pyk2, inflammation, carcinogenesis, metastasis, EMT

## Abstract

Focal adhesion kinase (FAK) and its homologous FAK-related proline-rich tyrosine kinase 2 (Pyk2) contain the same domain, exhibit high sequence homology and are defined as a distinct family of non-receptor tyrosine kinases. This group of kinases plays critical roles in cytoskeletal dynamics and cell adhesion by regulating survival and growth signaling. This review summarizes the physiological and pathological functions of Pyk2 in inflammation and cancers. In particular, overexpression of Pyk2 in cancerous tissues is correlated with poor outcomes. Pyk2 stimulates multiple oncogenic signaling pathways, such as Wnt/β-catenin, PI3K/Akt, MAPK/ERK, and TGF-β/EGFR/VEGF, and facilitates carcinogenesis, migration, invasion, epithelial–mesenchymal transition and metastasis. Therefore, Pyk2 is a high-value therapeutic target and has clinical significance.

## 1. Introduction

Focal adhesion kinases are coordinators transferring signals from integrins to downstream kinases, thus controlling cytoskeletal dynamics and cell adhesion by regulating survival and growth signaling, determining cell behavior [1]. The focal adhesion kinase family has two members: focal adhesion kinase (FAK) and proline-rich tyrosine kinase 2 (Pyk2). FAK often coordinates integrin-mediated cell migration in non-hematopoietic cells [2,3], however, it is not clear whether FAK plays a role in activation of adhesion- or cytoskeleton-dependent functions in human granulocytes [4,5]. Pyk2 is highly expressed in the central nervous system, epithelial cells and hematopoietic cells [6]. Numerous studies have demonstrated that FAK and Pyk2 have distinct roles. For instance, genetic deletion of FAK in mice causes mesoderm development defect and is embryonic lethal [3]. In contrast, loss of Pyk2 in mice does not result in obvious impairment in the development [7,8], but affects cell migration of macrophages and marginal zone B cells [7,8]. An increasing number of studies have demonstrated that Pyk2 has physiological and pathophysiological functions, for examples, in inflammation and cancers. 

## 2. Biological Functions of Pyk2

### 2.1. Cloning and Characterization of Pyk2

**Figure 1:**
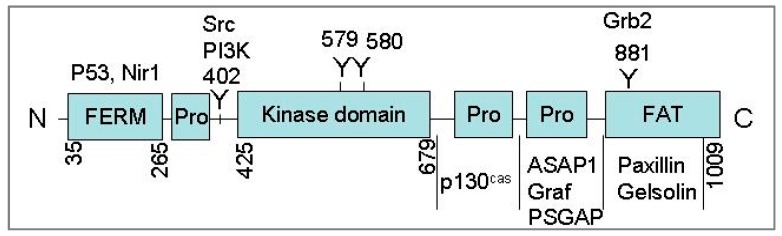
The main structure domains of Pyk2. Sites of tyrosine phosphorylation (402, 579, 580, and 881), amino acid positions for main domains and Pyk2 binding partners are also indicated.

Pyk2 was independently cloned by three research groups in 1995 [9,10,11]. Pyk2 is located at chromosome 8p21.2 and encodes a 110-kDa protein that shares a high degree of sequence similarity with FAK (48% amino acid identity and 65% similarity). However, the two proteins appear to be differentially regulated, which is attributed to differences in their C-terminal domains [6]. Pyk2 contains three functional domains: a NH_2_-terminal FERM (4.1/Ezrin/Radixin/Moesin) domain, a central kinase domain, and a C-terminal domain containing a focal adhesion targeting sequence, and three proline-rich sequences that mediate interactions with proteins containing SH3 domains (Figure 1).

Two splice isoforms of Pyk2 in the proline-rich sequences of the C-terminal region have been identified; one isoform contains an exon encoding 42 amino acids whereas the other isoform lacks this exon [12,13]. The short isoform is abundantly expressed in hematopoietic cells (and is referred to as Pyk2-H), whereas the un-spliced form is predominant in the brain. The second isoform described for Pyk2 lacks both the N-terminal FERM domain and the kinase domain, and is thus termed Pyk2-related non-kinase (PRNK); it consists of the C-terminal 228 residues of Pyk2 fused to nine unique N-terminal amino acids [12]. The structure of PRNK is similar to that of FAK related non-kinase (FRNK), the autonomously expressed C-terminal domain of FAK [14] suggesting that it may act as an endogenous inhibitor of Pyk2 activity in certain tissues. Adenovirus-mediated overexpression of PRNK prevented myocardial fibrosis in a rat model by inhibiting the phosphorylation of full-length Pyk2 [15]. Macrophages treated with a cell permeable TAT fusion protein containing the C-terminus of Pyk2 (TAT-PRNK) showed significantly impaired CD11b/CD18-mediated phagocytosis [16]. Stable PRNK-expressing squamous carcinoma cells exhibited low viability, low migratory ability, low invasive ability and low adhesion capacity [17]. PRNK does not interact with the Pyk2 partner p130^Cas^ or Graf, suggesting that its capacity to act as an endogenous regulator of Pyk2 may be restricted to certain cell types [12]. FRNK is a cytoskeletal regulatory protein. Recent studies have shown that reduced expression of FRNK prevented metastatic adhesion of HCC cells in a mouse model [18,19]. Moreover, FRNK negatively regulates IL-4-mediated inflammation by blocking eosinophil accumulation and transmigration via repressing STAT6-induced VCAM-1 and CCL26 expression at transcriptional and translational levels [20].

### 2.2. Cellular Functions of Pyk2

Pyk2 functions in the regulation of the actin cytoskeleton and cell polarization, adhesion, spreading and migration. Pyk2 is required for macrophage polarization and migration towards sites of inflammation [7,21]. Pyk2-deficient macrophages showed a delay in the formation of leading edge lamellipodia in response to chemokine stimulation and failed to detach from a substrate at the trailing edge resulting in drastically reduced migration [7]. Pyk2 contributes to complement mediated phagocytosis, but is dispensable for Fcγ receptor (FcγR)-mediated uptake [16]. Interestingly, the differential expression of *Yersinia pseudotuberculosis* adhesins determines the requirement for FAK and/or Pyk2 during bacterial phagocytosis by macrophages [22] , and this may be the result of distinct binding to surface integrins expressed on macrophage [23].

The effect of Pyk2 on lymphocyte function is especially important because Pyk2 is mainly expressed in cells of hematopoietic origin; Pyk2 plays a role in the regulation of the humoral immune response and must be expressed at a certain level for splenic B-cell differentiation and migration [8]. Both Pyk2 and FAK are important for CXCL13- and S1P-induced migration of B-2 cells and marginal zone B cells [24], in contrast, CD11a-mediated adhesion requires only Pyk2 whereas Akt-mediated pro-survival signaling requires only FAK. Although the deficiency of Pyk2 can cause a specific loss of short-lived effector CD8 T cells, loss of Pyk2 does not affect the development of memory-precursor CD8 T-cells [25]. Pyk2 contributes to cytotoxic T lymphocyte migration by regulating detachment of cells at the trailing edge [26]. In neutrophils, Pyk2 is required for integrin-mediated degranulation and migration, but Pyk2 is not involved in superoxide production [27]. In eosinophils, Pyk2 is not required for integrin-mediated adhesion, but is essential for β2 integrin-mediated spreading and migration [28]. Pyk2 is involved in normal bone remodeling [29,30,31]. *Pyk2*^−/−^ mice exhibit increased bone mass due to increased bone formation, resulted from enhanced osteoprogenitor cell differentiation and activation [31]. Furthermore, Pyk2 promotes osteoclastic bone resorption [32,33]. Pyk2 can also regulate cell–cell junctions. For instance, Pyk2 controls disassembly of vascular endothelial (VE)-cadherin-mediated cell-cell junctions and facilitates trans-endothelial migration of leukocytes [34]. In neuronal cells, Pyk2 is involved in regulating synaptic plasticity [35,36].

### 2.3. Pathological Functions of Pyk2

Pyk2 plays an important role in inflammatory diseases. For instance, Pyk2 controls inflammatory cell migration in vitro, and regulates allergic airway inflammation, cytokine secretion, and hyperresponsiveness in a mouse model of asthma [37]. Pyk2 inhibition also prevents neutrophil mediated acute lung injury without blocking neutrophil chemokines MIP2 and KC [38]. In addition, Pyk2/FAK inhibitor PF-562271 has shown reduced monosodium urate-mediated peritonitis of an NLRP3 activation model [39].

A recent study has also shown that ASC, an inflammasome adaptor protein that plays a role in the innate immune response and inflammatory diseases via self-oligomerization, could be regulated by FAK and Pyk2 via activating NLRP3 inflammasome. Pyk2, but not FAK, directly phosphorylates ASC, and only ASC phosphorylated at Tyr146 is involved in speck formation and interleukin (IL)-1β secretion. The inhibition of Pyk2 with small interfering RNA or inhibitors significantly abrogated ASC oligomerization, caspase-1 activation and IL-1β secretion [39]. A recent study showed that ASC specks released by microglia can bind to amyloid-β and increase amyloid-β oligomer formation and aggregates [40]. It is known that the deposition of amyloid-β is a hallmark of Alzheimer’s disease, and deposition of amyloid-β is accompanied by activation of the innate immune system and involves inflammasome-dependent formation of ASC specks in microglia. This finding is supported by an experiment in the transgenic double-mutant APPSwePSEN1dE9 mouse model, in which intrahippocampal injection of ASC specks resulted in spreading of amyloid-β, and administration of anti-ASC antibody abrogated amyloid-β accumulation and pathology [40]. Therefore, the seeding and spreading of amyloid-β pathology in Alzheimer’s disease is linked to inflammasome activation, and this linkage could be targeted by Pyk2.

*Pyk2^−/−^* mice exhibited increased bone mass in comparison with the *Pyk2^+/+^* mice [31,32]. In a postmenopausal osteoporosis rat model, loss of Pyk2 preserved bone density by enhancing bone formation without affecting bone resorption [31]. In contrast, Gil-Henn et al. found increased bone mass in *Pyk2^−/−^* mice, which might be associated with dysfunction of osteoclasts [32] because of insufficient formation of sealing zones and decreased bone resorption in *Pyk2^−/−^* mice. The differences seen between these two studies might be attributed to the age of the mice used for the experiments. These findings strongly suggest the importance of Pyk2 in bone homeostasis, implicating Pyk2 as a potential target in osteoporosis therapy.

Pyk2 is also highly expressed in forebrain neurons, especially in the hippocampus [9]. *Pyk2*^−/−^ mice exhibited several malfunction of hippocampal-related learning [41]. Pyk2 levels are decreased in the hippocampus of patients with Huntington disease. Normalizing Pyk2 expression in the hippocampus of R6/1 mice was able to rescue memory defects, spine pathology and PSD-95 distribution. Therefore, Pyk2 deficiency appears to contribute to cognitive impairments in Huntington’s disease.

## 3. Signaling Mechanisms by Which Pyk2 Regulates Inflammation

### 3.1. Pyk2 in Integrin Mediated Migration

It is well known that Pyk2 regulates cell migration. The regulation of cells involves three steps: first, location of migration; second, the cells require protrusive activity for migration; third, the cells should retract the trailing edge in order to migrate. Theoretically, Pyk2 has functions necessary to prepare the cells for this three-step migration. Pyk2 controls cell polarization; macrophages without Pyk2 expression are unable to establish a polarized morphology in response to stimulus [7]. Pyk2 also controls macrophage mobility, *Pyk2*-null macrophages exhibit impaired migration [7]. Moreover, inhibition of Pyk2 expression in macrophages attenuates colony-stimulating factor-1(CSF-1)-induced cell invasion [22]. Similar functions of Pyk2 have been observed in osteoclast cells [42], cytotoxic T cells [26], marginal zone B cells [43], eosinophils [28], neutrophils [27,44] and differentiated HL60 cells [45].

The regulation of cell migration by Pyk2 initially involves disassembly of focal adhesions, followed by extension of the leading edge and retraction of the trailing edge [46]. Pyk2 is activated by an increase in the cytoplasmic concentration of free Ca^2+^, which could be caused by a variety of extracellular stimuli, such as adhesion ligands and stress signals (e.g., tumor necrosis factor alpha, [TNFα]) [6]. After stimulation, Pyk2 undergoes auto-phosphorylation at Tyr402, leading to activation. Next, Pyk2 recruits Src family kinases (SFK) and stimulates their activities, leading to further phosphorylation of Pyk2 at three other tyrosine sites (Tyr579, Tyr580 and Tyr881). Among these three tyrosine sites, Tyr579 and Tyr580 are more important because phosphorylation of these two tyrosines is required for full activation of Pyk2. Pyk2 functions at the cross point of integrins and G protein-coupled receptor (GPCR) signaling [47]. Pyk2 can be translocated to focal adhesions by extracellular matrix proteins and by activation of GPCR. This translocation can further enhance Pyk2 phosphorylation. Pyk2 phosphorylation determines localization; for example, Pyk2 phosphorylation at Y579 and Y580 localizes to the leading edge, whereas Pyk2 phosphorylated at Y881 is mostly localized at the trailing edge.

Activation of the RhoA-ROCK signaling pathway is crucial for the detachment of migrating cells. Pyk2 deficiency leads to impaired detachment of the rear end of migrating cells [7,26]. Recent studies also found that Pyk2 activation mediated by the integrin LFA1 generates LAT-GRB2-SKAP1 complexes, and thus terminates T-cell adhesion to dendritic cells [48]. RhoA activation can enhance activities of some guanine nucleotide exchange factors (GEFs), including p190Rho, p115Rho, LARG, and GEF-H1. Pyk2 facilitates RhoA activation and migration by promoting p190RhoGEF expression in *FAK*^−/−^ fibroblast cells [49].

Cell migration is also affected by changes in the actin cytoskeleton that are regulated by Pyk2-mediated activation of Rho and Cdc42 via a reduced GTP hydrolysis activity toward Rho and Cdc42 [50], although the underlying mechanism is unclear.

### 3.2. Pyk2 for Cytokine Secretion

Activated macrophages secrete pro-inflammatory cytokines to recruit other immune cells to the site of inflammation. Pyk2 regulates secretion of the pro-inflammatory cytokines IL-1β and IL-18 from macrophages [51]. A recent study found that Pyk2 phosphorylates the inflammasome adaptor protein ASC at Tyr146, and the phosphorylated ASC then participates in speck formation that leads to caspase-1 activation and secretion of IL-1β and IL-18 [39]. The role of Pyk2 in T cell activation and cytokine secretion has not been fully elucidated [52].

### 3.3. Pyk2 for Cell Survival

Increased Pyk2 levels are observed in *FAK* knockout fibroblasts and induce an intrinsic mechanism to increase cell survival [53], partially by nuclear translocation and selective regulation of the tumor suppressor p53 by Pyk2. The Pyk2 FERM domain has the capability to promote Mdm2-dependent p53 ubiquitination. Thus, Pyk2 facilitates cell growth and survival via the Pyk2-Mdm2-P53 axis and in a kinase-independent manner.

## 4. Pyk2 and Cancers

Numerous studies have demonstrated increased Pyk2 expression and activation in cancers of the lung, breast, gastrointestinal tract, prostate, and multiple myeloma, compared to normal tissues. Functional studies have shown that increased expression of Pyk2 promotes cancer cell proliferation, migration, invasion and metastasis, and either knockdown or pharmacological inhibition of Pyk2 represses malignant characteristics of cancer cells. Therefore, Pyk2 could be a therapeutic target for cancers.

### 4.1. Pyk2 and Lung Cancer

It is reported that levels of Pyk2 mRNA and protein and the phosphorylated PYK2 form pY881 are increased in non-small cell lung cancer (NSCLC) lesion compared with matched non-cancerous tissues, and that Pyk2 and Pyk2 pY881 are independent prognostic factors for patients with NSCLC [54]. Another study showed that Pyk2 expression is upregulated in NSCLC and correlated with higher metastatic potential [55]. In vitro studies showed that increased expression of Pyk2 in human lung cancer cells upregulated the expression of ALDH1a1, ABCG2 and Bmi-1, and enhanced colony formation in soft agar [54], while knockdown of Pyk2 expression inhibited anchorage-independent survival and proliferation of NSCLC cells [56].

Suppressor of cytokine signaling 3 (SOCS3) is a suppressor of cytokine responses and plays a negative role in cell migration. In vitro experiments showed that SOCS3 expression was found to be silenced in NSCLC due to hypermethylation, and Pyk2 activity was increased. However, reactivation of SOCS3 attenuated Pyk2 expression and phosphorylation, which in turn, promoted apoptosis and inhibited cell proliferation, migration and invasion, suggesting an inverse correlation for expression and biological functions between Pyk2 and SOCS3 in NSCLC [57,58].

### 4.2. Pyk2 and Breast Cancer

The association between Pyk2 and breast cancer has been well studied. Pyk2 expression is significantly increased in early and advanced breast cancer and co-overexpressed with ErbB-2 in early stages of ductal in situ carcinoma and invasive breast cancer [59]. It has been reported that Pyk2 is a key effector of EGFR and HER2 signaling in breast cancer, in which Pyk2 is activated by EGF and heregulin (HRG), and positively regulates EGF/HRG-induced cell migration and invasion. Importantly, loss of Pyk2 leads to reduced transcription of matrix metalloproteinase 9 (MMP9) and cytokine IL-8induced by EGF/HRG, while IL-8 inhibition abrogates EGF-induced MMP9 transcription and suppresses cell invasion [60]. IL-8 is transcriptionally regulated by signal transducer and activator of transcription 3 (STAT3) and induces Pyk2 activation and prolongs the EGF-induced the phosphorylation of PYK2, STAT3 and ERK1/2. Therefore, Pyk2 is a common downstream effector of ErbB and IL-8 receptors and coordinates these signaling pathways through a positive feedback loop, hence, enhancing cancer cell invasion [60].

Chemokine (C-C motif) ligand 18 (CCL18) is derived from tumor-associated macrophages and has shown an ability to facilitate breast cancer metastasis. A study showed that Pyk2 and Src play important roles during CCL18-induced breast cancer metastasis [61]. It is known that CCL18 initiates Pyk2 and Src phosphorylation leading to breast cancer metastasis via its functional GPCR PITPNM3. Binding of CCL8 to PITPNM3 leads to translocation of Pyk2 from the cytoplasm to the plasma membrane and formation of a stable complex with PITPNM3 that subsequently activates Src kinase. Furthermore, CCL18 stimulates Pyk2 and Src, initiating integrin alpha5/β1 clustering-dependent adherence, migration, and invasion [61].

Additional studies have shown that CCL18-induced breast cancer metastasis occurs through the Pyk2 receptor, Pyk2 N-terminal domain interacting receptor 1 (Nir1), and that Nir1 expression is associated with lymph nodes and distant metastasis in patients with invasive breast cancer [62]. Nir1 first binds to CCL18 and then promotes the phosphorylation of Akt, LIN-11 and LIMK (Isl1 and MEC-3 protein domain kinase). Interestingly, the binding between Nir1 and CCL18 could cause cell mesenchymal morphology and lead to epithelial–mesenchymal transition (EMT). Mechanistic studies revealed that the binding of Nir1 and CCL18 stabilized Snail through the Akt/GSK3β (glycogen synthase kinase 3β) signaling pathway [62].

It has been also reported that Pyk2 expression is significantly upregulated in recurrent human breast cancers, and that differential expression of Pyk2 in human MDA-MB-231 breast cancer cells is predictive for metastasis, but not for invasion [63]. In addition, transforming growth factor-β (TGF-β) stimulated Pyk2 expression and robustly upregulated their expression of Pyk2 in metastatic human and murine breast cancer cells during EMT programs. In contrast, reduction of Pyk2 activity through genetic engineering and pharmacological inhibition abrogated the ability of breast cancer cells to form orthotopic mammary tumors and undergo invasion, and inhibited the metastatic outgrowth of disseminated breast cancer cells in the lungs of mice. Mechanistic studies revealed that Pyk2 expression was negatively correlated with the expression of E-cadherin, and that elevated Pyk2 levels stabilized β1 integrin expression, initiated EMT programs and facilitated the metastatic cascade regulated by TGF-β [63].

Pyk2 is also reported to be a regulator of mammary cancer stem cells (MaCSCs), rare populations of cells that are capable of self-renewal to drive mammary tumorigenesis and metastasis [64]. Pyk2 compensated for FAK function in mammary tumor cells isolated from *FAK^−/−^* mice [64]. Increased expression of Pyk2 was found in pulmonary metastatic nodules of the mammary cancer mouse model, and inhibition of Pyk2 significantly inhibited mammary tumor formation and metastasis. Interestingly, Pyk2 was up-regulated in MaCSCs, but not in the general mammary tumor cells of primary tumors developed in mammary cancer mouse model, as metastasis is known to be driven by MaCSCs. Moreover, inhibition of Pyk2 in FAK-null MaCSCs dramatically suppressed cancer cell sphere formation and migration in vitro as well as self-renewal, tumorigenesis, and metastasis in mice through the FAK-Pyk2-PI3K/Akt signaling pathway [64].

### 4.3. Pyk2 and Gastrointestinal Cancer

It is well known that aberrant activation of Wnt/β-catenin signaling plays a critical role in colorectal carcinogenesis and progression, and a study reported that FAK/Pyk2 promotes the Wnt/β-catenin pathway and colorectal carcinogenesis via a novel FAK/PYK2/GSK3β(Y216)/β-catenin regulation axis by phosphorylating GSK3β [65]. The authors found that FAK and Pyk2 expression levels were significantly increased in human colorectal cancers and in the adenomas of *ApcMin*/+ mice. They further showed that FAK and Pyk2 promoted the Wnt/β-catenin pathway by phosphorylating GSK3β(Y216), resulting in -β-catenin accumulation and its translocation from the cytoplasm to nuclei and gastrointestinal tumor formation, as Wnt-stimulated β-catenin accumulation requires Wnt-induced GSK3β/β-transducing repeats-containing proteins (β-TrCP) interaction. Interestingly, chemical inhibitor-induced repression of FAK/Pyk2 suppressed intestinal adenoma development in *ApcMin/+* mice, in which intestinal levels of phospho-GSK3β(Y216) and β-catenin were dramatically downregulated, suggesting that the FAK/Pyk2/GSK3β(Y216) axis is critical for Wnt/β-catenin signaling activation in Apc-mutation-induced colorectal carcinogenesis and progression [65]. This regulation of Pyk2 and GSK3β was supported by a recent study on the differentiation of human neuronal progenitor cells (hNPCs), in which Wnt3a activates Pyk2, and subsequently regulates GSK3β phosphorylation and β-catenin stabilization, suggesting that Pyk2 plays an important role in coordinating the stabilization of β-catenin in the crosstalk between Wnt/β-catenin and Wnt/Ca2+ signaling pathways upon Wnt3a stimulation in differentiating hNPCs [66].

### 4.4. Pyk2 and Liver Cancer

The expression levels of Pyk2 and FAK were also found to be elevated in liver cancers, compared with their adjacent non-tumor tissues, and the elevated expression of Pyk2 and FAK was significantly correlated with shorter survival times and poor outcomes [67]. In addition, higher expression of Pyk2/FAK was positively correlated with larger tumor size and advanced Edmonson grading. In vivo studies further showed that infiltrative tumor cells and lung metastatic tumor foci exhibited higher expression of Pyk2.

Moreover, overexpression of Pyk2 in human hepatocellular carcinoma (HCC) cell lines resulted in an increase of cell proliferation, colony formation and invasion, and accelerated wound healing by stimulating actin stress fiber polymerization in vitro [68]. Mechanistic studies indicated that increasing the expression of Pyk2 facilitated c-Src activation, and that the Pyk2/c-Src complex triggered the activation of the extracellular signal-regulated kinase (ERK)/mitogen-activated protein kinase (ERK/MAPK) signaling pathway, suggesting that Pyk2-mediated cell proliferation and invasiveness occurred through aberrant activation of the c-Src and ERK/MAPK-signaling pathways [68].

Interestingly, a recent study showed that high expression of Pyk2 in peritumoral tissues was associated with poor survival, disease recurrence, and distant metastasis in HCC [69], and a higher Pyk2 density in both tumor and peritumoral tissues was associated with serum alpha-fetoprotein (AFP) levels. However, only higher peritumoral Pyk2 density was correlated with lower disease-free survival, vascular invasion and VEGF expression, which was mediated through the a2-activated PI3K-AKT pathway during HCC progression and invasion.

Clinical and experimental studies have demonstrated that microRNAs (miRNAs) play important roles during carcinogenesis and progression in various cancers, which is through targeting downstream genes. Bioinformatic studies and data mining have identified that Pyk2 is a target of miR-23b, miR-517a and miR-517c [70,71]. Experimental and functional studies showed that miR-23b expression was significantly downregulated in HCC tissues and positively correlated with intrahepatic metastasis of HCC, which was linked to the upregulation of Pyk2. Moreover, inhibition of metastasis by miR-23b could be restored by increasing the expression of Pyk2 [70]. In addition, ectopic expression of miR-517a and miR-517c was sufficient to inhibit cancer cell proliferation by blocking the G2/M transition, in contrast, knockdown of miR-517a and miR-517c expression in the HCC cells facilitated cell proliferation in vitro and tumor growth in vivo also through targeting Pyk2 [71].

### 4.5. Pyk2 and Other Cancers

A previous study reported that Pyk2 plays a central role in the migratory behavior of glioblastomas [72], and another study showed that Pyk2 promotes the migration and invasion of glioma cells and that the inhibition of Pyk2 attenuates glioma progression [73]. MicroRNA expression profiling in migrating glioblastoma cells revealed that tumor suppressive miR-23b inhibited glioma cell migration and invasion by targeting Pyk2 [74]. In medulloblastoma cells, tyrosine kinase receptor c-Met was found to induce phosphorylation of FAK and Pyk2, which in turn, mediated the malignant effects of c-Met on medulloblastoma cell proliferation, migration, and invasion [75].

Patients with multiple myeloma patients have higher expression of Pyk2, in comparison to healthy subjects. Pyk2 inhibition delayed tumor growth of multiple myeloma in vivo, and reduced cell proliferation, cell-cycle progression, and adhesion in vitro. In contrast, increasing expression of Pyk2 promoted tumor growth and shortened survival time through Wnt/β-catenin signaling by Pyk2-mediated stabilization of β-catenin and activation of c-Myc and Cyclin D1, the direct downstream targets of β-catenin [76].

Multiple myeloma is one of the incurable malignancies, in part, due to the effects of bone marrow microenvironment on therapeutic response. Amplification and activation of STAT3 plays an important role in β1 integrin-mediated adhesion to fibronectin and IL-6 signaling, and the STAT3- β1 integrin-IL-6 axis could be regulated by FAK/Pyk2. Inhibition of Pyk2 by chemical inhibitors and small molecules reduced STAT3 phosphorylation and amplification. Co-culture of multiple myeloma cells with patient bone marrow stromal cells (BMSCs) promoted Pyk2-STAT3 signaling, similar to the effects resulting from β1 integrin stimulation. Specific inhibitor of Pyk2 or small interfering RNA targeting Pyk2 promoted cell death and repressed colony formation in BMSC-adherent myeloma cell lines. A reduction in Pyk2 also delayed multiple myeloma progression [77].

A study on head and neck metastatic squamous cell carcinomas showed that chemokine receptor 7 could accelerate cancer cell migration and invasion via the RhoA/ROCK-Pyk2 signaling pathway [78]. Pyk2 was also shown to be significantly overexpressed and a prognostic factor in ovarian cancer, and interaction of Pyk2 with CCL18 promoted ovarian cancer cell migration [79].

### 4.6. Pyk2 in Cancer Therapy

The focal adhesion tyrosine kinases FAK and Pyk2 are crucial mediators for the activation of signaling pathways from cell surface growth factors and adhesion receptors to cell proliferation, migration, and survival. Increasing evidence has demonstrated that FAK/Pyk2 signaling regulates hematopoietic cell differentiation, bone mass formation, neuronal degeneration, inflammatory response and cancer. Therefore, Pyk2 is a valuable therapeutic target for inflammatory diseases and malignancies [80,81]. Based on protein structure, targeting tyrosine kinases has focused on the ATP binding pocket to inhibit catalytic activity and repress the pathways involved in carcinogenesis, EMT and metastasis. Although there are no specific inhibitors to date, several catalytic inhibitors of the focal adhesion kinases have been developed. TAE226, a bis-anilino pyrimidine compound, is an ATP competitive inhibitor that inhibits FAK kinase activity. TAE226 can also inhibit Pyk2 activity. Treatment of cultured glioma cell lines or ovarian cancer cell lines with TAE226 inhibited cell proliferation and increased apoptosis [82,83]. Another ATP competitive inhibitor of FAK and Pyk2 is PF-562,271, a methane sulfonamide diaminopyrimidine. Cell-based assays demonstrated significant selectivity for FAK and Pyk2 over a panel of related kinases. Administration of PF-562,271 significantly inhibited cancer cell growth in xenograft tumor models, showing robust antitumor effects [84]. An alternative approach of developing non-catalytic inhibitors to the inhibition of kinase activity is to target protein–protein interactions that play a role in the regulation of kinase activity, in order to achieve targeting specificity [85,86]. Several new ligands have been reported to bind and inhibit kinase function through an allosteric mechanism. Studies have demonstrated that the FERM domain of Pyk2 is critical for Pyk2-stimulated migration of glioma cells [73,87].

The immunosuppressive tumor microenvironment is critical to the treatment of pancreatic ductal adenocarcinoma (PDAC). However, a recent study using a selective FAK inhibitor VS-4718 significantly attenuated tumor progression, leading to the longer survival time of the PDAC mouse model by reducing tumor fibrosis and decreased numbers of tumor-infiltrating immunosuppressive cells [88]. In addition, a second-generation inhibitor VS-6063 (also known as defactinib or PF-04554878) of FAK/PYK2 has been used for phase I trial in Japanese patients with advanced solid tumors [89]. In ovarian cancer cells, administration of etoposide phosphate (VP-16) could promote SKOV3 cell apoptosis, but VP-16-induced repopulation effects were partially reversed by the FAK inhibitor PF562271 [90]. A recent study reported that two small molecule inhibitors PF-573228 and PF-431396, dual specificity inhibitors of FAK and PYK2, as well as another small molecule inhibitor VS-6063 (specifically inhibits FAK but not PYK2), could inhibit cancer cell growth and anchorage-independent colony formation and induce apoptosis and cell cycle arrest [91]. Moreover, PF-573228 decreased tumor organoid size and increased organoid cell death in the 3D-tumor organoids [91]. As to immunotherapy, a study has shown that Pyk2 regulates the ability of T-cells to react against foreign antigens and cancers, suggesting the potential of Pyk2 inhibitors to increase T-cell conjugation in anti-tumor immunotherapy [48].

## 5. Conclusions and Perspective

**Figure 2:**
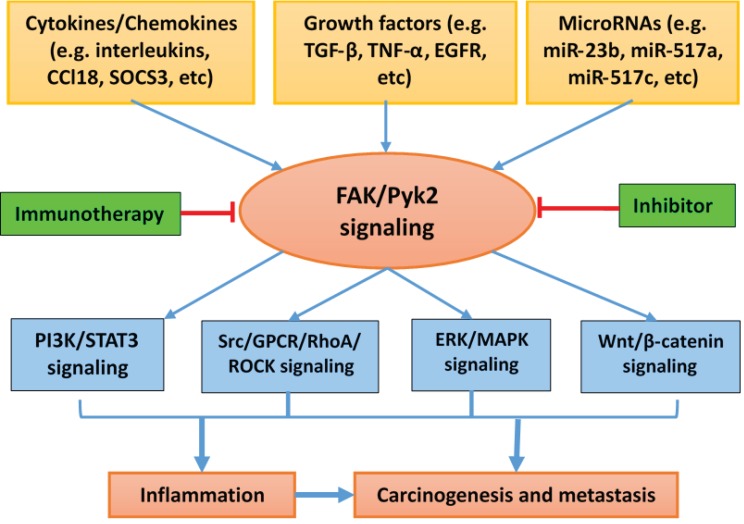
Scheme of the regulation and interaction of FAK/Pyk2 signaling in inflammation and cancer.

Proline-rich tyrosine kinase 2 (Pyk2) has important physiological and pathological roles in regulating inflammatory and oncogenic signaling pathways and is functionally involved in inflammation and cancers, including the process of carcinogenesis, epithelial–mesenchymal transition, and metastasis. Therefore, Pyk2 represents a high-value therapeutic target. (Figure 2). Complete understanding of Pyk2-mediated mechanisms during cancer formation and progression and development of selectively specific inhibitors for Pyk2 will have clinical translational significance.

## Abbreviations

FAK	focal adhesion kinase
Pyk2	proline-rich tyrosine kinase 2
CSF-1	colony-stimulating factor-1
TNFα	tumor necrosis factor alpha
GPCR	G protein-coupled receptor
NSCLC	non-small cell lung cancer
SOCS3	suppressor of cytokine signaling 3
CCL18	chemokine (C-C motif) ligand 18
EMT	epithelial-mesenchymal transition
TGF-β	transforming growth factor-β
MaCSCs	mammary cancer stem cells
GSK3β	glycogen synthase kinase 3β
HCC	hepatocellular carcinoma
ERK	extracellular signal-regulated kinase
MAPK	mitogen-activated protein kinase
STAT3	signal transducer and activator of transcription 3
